# Sclérodermie localisée: à propos de 24 cas

**DOI:** 10.11604/pamj.2018.29.53.11592

**Published:** 2018-01-19

**Authors:** Sara Elloudi, Hanane Baybay, Salim Gallouj, Fatima Zohra Mernissi

**Affiliations:** 1 Service de Dermatologie-Vénérologie, CHU Hassan II, Route Sidi Hrazem, Fès, Maroc

**Keywords:** Morphée, sclérodermie localisée, pronostic, Morphea, localized scleroderma, prognosis

## Abstract

La morphée ou sclérodermie localisée est définie par un état scléreux de la peau pouvant s’étendre aux tissus sous-cutanés mais sans phénomène de Raynaud ni atteinte viscérale. Certaines formes cliniques peuvent avoir un retentissement fonctionnel et esthétique, d’où l’intérêt d’une prise en charge précoce au stade inflammatoire. Nous décrivons les caractéristiques épidémio-cliniques, thérapeutiques et évolutives des différentes formes de morphée à travers une série de 24 cas.

## Abstract (en)

Morphea, also known as localized scleroderma, is defined as a sclerosus skin condition that can extend to the subcutaneous tissue but with no Raynaud’s phenomenon or visceral involvement. Some clinical forms may have a functional and aesthetic impact, hence the interest of treatment of early stage inflammation. We here describe the epidemio-clinical, therapeutic and evolutionary features of different forms of morphea, by reporting a series of 24 cases.

## Introduction

La morphée ou sclérodermie localisée est définie par un état scléreux de la peau pouvant s’étendre aux tissus sous-cutanés mais sans phénomène de Raynaud ni atteinte viscérale. Son évolution est imprévisible, mais elle n’engage presque jamais le pronostic vital. Le but de notre travail est d’étudier les caractéristiques épidémio-cliniques, thérapeutiques et évolutives des morphées.

## Méthodes

Etude rétrospective descriptive sur une période de 4 ans (Janvier 2012- Janvier 2016) portant sur les patients suivis au service de dermatologie du CHU Hassan II de Fès pour une morphée. Le diagnostic était porté sur l’aspect clinique et ou l’étude histologique.

## Résultats

**Figure 1:**
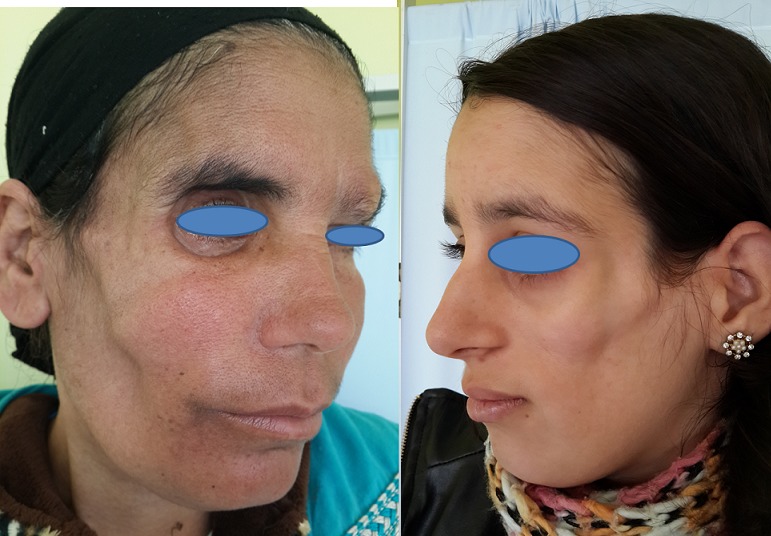
Atrophie hémifaciale de Parry et Romberg

**Figure 2:**
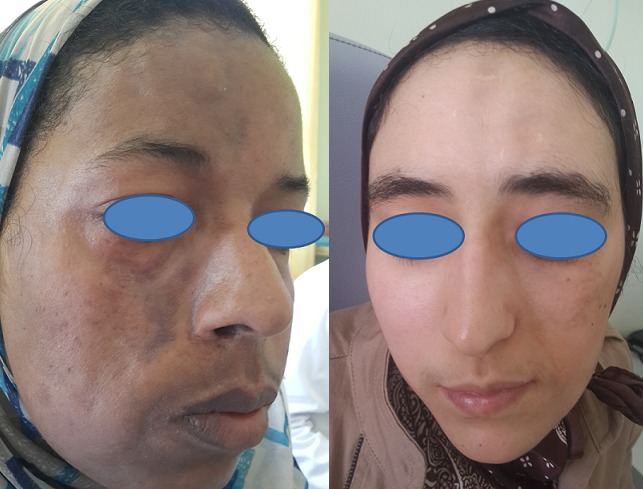
Morphée en coup de sabre

**Figure 3:**
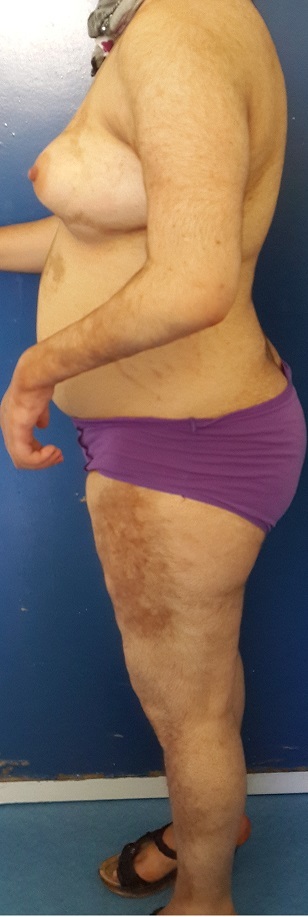
Morphée hémicorporelle

**Figure 4:**
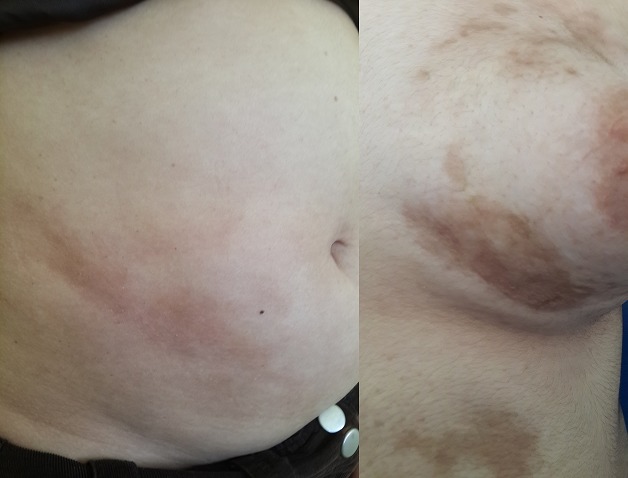
Morphée en plaque

**Figure 5:**
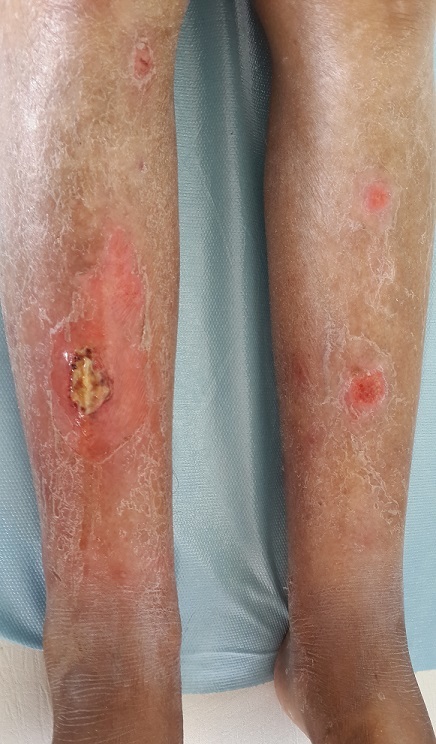
Morphée bulleuse et fasciite de Shulman

Sur une durée de 4 ans, 24 cas de morphèe ont été colligés. Le sexe ratio était de 3F/1H. L’âge moyen de nos patients était de 35,37 ans (4 à 62 ans). Le délai moyen de diagnostic était de 29 mois. Un facteur déclenchant ou aggravant était retrouvé chez 6 patients (25 %). La morphée était de type linéaire dans 12 cas (50 %): monomélique et atrophie hémifaciale de Parry et Romberg dans 4 cas chacun (Figure 1), en coup de sabre dans 2 cas (Figure 2), dimélique et hémicorporelle dans 1 cas chacun (Figure 3), et en plaque dans 9 cas (37,5%) (Figure 4), dont un cas était de type atrophodermie de Pasini-Pierini. 3 patients avaient une association de deux type en même temps: en bande et en plaque dans 2 cas chacun, forme bulleuse et fasciite de shulman dans un cas (Figure 5). Le nombre, la taille ainsi que le siège des lésions étaient variables, en fonction du type de morphée, ainsi, la forme linéaire siégeait essentiellement au niveau des membres et visage, alors que le type en plaque se localise au niveau du tronc et de l’abdomen. Des affections dysimmunitaires ou inflammatoires étaient associées (32%): thyroïdite auto-immune dans 4 cas, syndrome de Gougerot Sjögren, pelade universelle, dermatomyosite et dermatite atopique dans un cas chacun. La sérologie borélienne négative était réalisée dans 17 cas. Seulement 2 patients avaient un bilan immunologique positif. La biopsie cutanée confirmait le diagnostic dans 17 cas. L’IRM a confirmé l’extension en profondeur de morphée dans 9 cas. La durée moyenne de suivi était de 2 ans. 4 malades ont nécessité un traitement local seul (dermocorticoïde classe forte et calcipotriol ou tacrolimus topique 0,01%), 18 ont bénéficié d’un traitement général associé (corticoïdes associé au Méthotrexate ou Colchicine). L’évolution était jugée favorable. Des séquelles fonctionnelles et esthétiques étaient observées dans 11 cas (morphée en bande; en regard d’une articulation; et morphée en coup de sabre et Parry et Romberg).

## Discussion

La morphée est une affection rare, avec une incidence estimée à 27 cas par million d’habitants [1]. Sa physiopathogénie est toujours mal connue. Une origine auto-immune a été suggérée [1, 2], et certains facteurs de risque ont été incriminés tel que le traumatisme, l’irradiation, l’infection à *Borrelia burgdorferi*, ou certains médicaments par leur effet profibrotique, ischémique ou toxique [3]. Dans notre série, l’association à une affection auto-immune (32%), et la présence d’un facteur déclenchant (25%) approuvent ces probabilités. Cependant, on n’a pas trouvé d’association à un agent infectieux notamment à *Borrelia burgdorferi*. La morphée peut prendre différents aspects cliniques comme l’illustre notre série. Sur le plan clinique, la forme en plaque est la plus fréquente retrouvée dans 56% dans l’étude de Peterson [4], alors que dans notre série, la forme linéaire était la plus retrouvée (50% des cas), dont 24% étaient des enfants. Le traitement et le pronostic de la morphée dépendent de la forme clinique et du délai de consultation [5]. Ainsi, les type en plaques et superficielles avaient évolué favorablement sous traitement local. Par contre, des séquelles fonctionnelles et esthétiques étaient observées dans les formes profondes qui nécessitaient un traitement par voie générale. Paradoxalement, l’évolution était favorable sous corticothérapie orale et colchicine dans le cas de la fasciite de Shulman associée à la morphée bulleuse.

## Conclusion

Bien que le pronostic des sclérodermies localisées soit généralement bon, certaines formes cliniques peuvent avoir un retentissement fonctionnel et esthétique surtout au niveau de la face et des membres en regard des articulations. Une corticothérapie par voie générale associée ou non à un traitement immunosuppresseur ou antifibrotique semble nécessaire pour les formes linéaires et profondes.

### Etat des connaissances actuelle sur le sujet

- La morphée est un état scléreux de la peau pouvant s’étendre aux tissus sous-cutanés mais sans phénomène de Raynaud ni atteinte viscérale;
- Il existe plusieurs formes cliniques en fonction de l’aspect clinique, de l’étendu et de l’extension en profondeur;
- Le pronostic est essentiellement fonctionnel et esthétique.

### Contribution de notre étude à la connaissance

- Il s’agit d’une pathologie rare et peu connu chez les non dermatologues;
- Description du profil épidemio-clinique, thérapeutique et évolutif des différentes formes cliniques dans notre contexte avec une riche iconographie;
- Une corticothérapie par voie générale associée à un traitement immunosupresseur ou antifibrotique semble nécessaire pour les formes linéaires et profondes.

## Conflits d’intérêts

Les auteurs ne déclarent aucun conflit d’intérêts.
